# Broader Implications of Modeling and Simulation (M&S) Tools in Pharmacotherapeutic Decisions: A Cautionary Optimism

**DOI:** 10.3389/fphar.2020.00571

**Published:** 2020-04-29

**Authors:** Ayyappa Chaturvedula, Brittany N. Palasik, Hae Jin Cho, Navin Goyal

**Affiliations:** ^1^Pharmacotherapy, System College of Pharmacy, University of North Texas Health Science Center, Fort Worth, TX, United States; ^2^Clinical Pharmacology, GlaxoSmithKline, Collegeville, PA, United States

**Keywords:** pharmacotherapy, pharmacometric models, pediatrics, geriatrics, precision dosing

## Introduction

While not an entirely new concept, emergence of *precision medicine* has been catalyzed by the National Institutes of Health (NIH)’s “Precision Medicine Initiative” (Collins and Varmus, 2015). This initiative has goals to understand how one’s genetics, environment, and lifestyle can help determine the best approach to prevent or treat disease. The “precision” concept was initially interpreted rather narrowly to the clinical implementation of pharmacogenomics, but the clinical community recognized the importance of broader application to all stages of clinical care: prevention, diagnosis, and treatment (Peck, 2018; Verstegen and Ito, 2019). Pharmacotherapy is an important component of treatment phase of the clinical care which aims to provide appropriate medicine and dose to each patient, which we now call “precision dosing.” Clinical pharmacologists and clinical pharmacists have long been applying such concept to drug dosing within the framework of “Therapeutic Drug Monitoring (TDM)” for narrow therapeutic index drugs.

Drugs are generally approved based on “average” improvement of disease outcomes in a population. There are a myriad of factors that contribute to variability in drug exposure and response including genetic polymorphisms in drug metabolism (e.g., CYP2D6, CYP2C19) and pharmacodynamic targets (e.g., HER+ve versus HER-ve), demographic differences (e.g., age, weight), drug-drug interactions, hepatic/renal dysfunction, and patient behaviors (e.g. adherence). The *response* here extends from drug-related efficacy to safety.

There has been a major shift in the drug development paradigm in the past few decades where the scientific focus shifted from “confirming” efficacy to “learning” drug exposure-response relationships (Sheiner, 1997). The most important enabler of this shift was the development of a pharmacostastical modeling methodology, nonlinear mixed effects modeling (NLME), that can analyze sparsely sampled exposure and response data (Sheiner and Beal, 1980; Sheiner and Beal, 1981; Sheiner and Beal, 1983). The original intent was to use the methodology to study patient populations by utilizing routinely collected TDM data.

Simplistically, a model is a set of mathematical expressions that characterize drug absorption, distribution, metabolism, and elimination. Any response, safety or efficacy, may be assumed to be a function of the drug exposure. Such models can be expanded to include characteristics related to the patient (e.g., age, weight, adherence, maturation, and/or polymorphisms of metabolic enzymes, sensitivity); drug (e.g., solubility, lipophilicity, potency); and disease (e.g., baseline severity, resistance). Advances in computational methodology and power has opened up the possibility of studying exposure-response relationships of new drugs in late phase clinical trials in actual patients where the blood sampling is very limited. The application of these modeling and simulation (M&S) tools in drug development evolved into the field of “pharmacometrics” which became a branch of clinical pharmacology. Model based approaches have been well accepted by regulatory agencies with the issuance of guidance documents on how to conduct and report such analyses (Wang et al., 2019). The use of quantitative clinical pharmacology principles has brought considerable efficiencies in drug development in many areas (dose, trial design, sample size, identifying appropriate patient population, etc.).

One may intuitively assume that the next logical step would be a comparable implementation of model-based approaches in clinical practice thereby bringing about “precision dosing” (Neely and Jelliffe, 2010). While this has not been the case, a considerable scientific progress has been made demonstrating improved patient outcomes and cost-effectiveness due to precision dosing (Vinks et al., 2020). This commentary discusses the need of adopting such tools for better pharmacotherapy in two of the most vulnerable populations, pediatrics, and geriatrics.

## Dosing in Pediatric Population

Optimizing pharmacotherapy in the pediatric population has its unique set of challenges. Historically, medications in pediatrics were often used off-label, based on limited pediatric evidence from case reports or small studies. In fact, before the enactment of the 1997 U.S. FDA Modernization Act, only about 30% of FDA approved medications were indicated for pediatrics (U.S. Department of Health and Human Services and Food and Drug Administration Center for drug evaluation and research, 2007). Lacking evidence in comparison to that of adults, pediatrics are more vulnerable to receiving subtherapeutic or supratherapeutic dosing of medications (Barker et al., 2018).

Moreover, the heterogeneous characteristics of pediatrics contribute to the challenges of medication dosing. The age of pediatric patients ranges from birth to 18 years old, encompassing neonates, infants, children, and adolescents. Particularly in neonates, there is an added component of gestational age and postmenstrual age to account for when dosing medications. The body weight and body surface area are factors that also have a wide spectrum within the population (Barker et al., 2018). Other developmental changes in pediatrics include, but are not limited to, metabolic capacity of enzymes such as the cytochrome P-450 isoforms (CYPs), distribution sites, gastrointestinal function, and renal function (Kearns et al., 2003). As such, the anatomic and physiologic development can vary vastly in pediatrics, posing as additional obstacles to optimizing pharmacotherapy in individual patients.

## Dosing in Geriatric Population

Individualized dosing and therapeutic decision-making for geriatric patients in clinical practice is of utmost importance. The geriatric population is the fastest growing population in the United States, largely due to the aging of the Baby Boom generation (United States Census Bureau, 2017). As this population continues to age, there is an increase in disease burden which results in polypharmacy, or the consumption of multiple medications. Polypharmacy can cause drug-drug interactions and adverse events in geriatric patients (Cho et al., 2011). Additionally, chronological age does not necessarily correlate with linear changes in pharmacokinetic parameters. For example, a frail patient may respond very differently to a similar medication dose as a strong patient who maintains most activities of daily living. High variability in response to medications is seen among geriatric patients (Charlesworth et al., 2015).

Geriatric patients are largely excluded in clinical trials, making it difficult to apply medication dosing recommendations to this population (Denson and Mahipal, 2014; Lau et al., 2015; Shenoy and Harugeri, 2015). Pharmacokinetic changes in elderly patients, such as increased body fat percentage and reduction in albumin, are clearly defined. However, there is minimal data describing and predicting pharmacodynamic changes in this population, such as alterations in receptor sensitivity (Gad, 2003). There is also minimal predictive dosing in geriatric patients in association with frailty, functional deficits of multi-organ systems, and hepatic drug metabolism. Hence, medication dose estimation for elderly patients has become a guessing game, in which clinicians are encouraged to “start low and go slow” (Charlesworth et al., 2015).

## M&S Tools

The exponential progress in computational power over the last decade has made transition of model-based approaches from bench to patient bedside relatively easy. There exist software tools that can be employed to predict drug-drug interactions and obviate the need for conducting resource intensive clinical trials. There are now mobile phone apps that can help health care providers continuously monitor the patient’s drug exposure and/or response and accordingly adjust treatment (dose, frequency) in real time. There are efforts in developing hospital wide systems and applications that integrate multisource data using such models (patient characteristics, comedication and prior drug efficacy and safety data) to recommend appropriate dosing regimen to health care providers for their patients. These models provide a valuable knowledge link between clinical trial information and clinic-based dosing. They may be built with data from multiple sources (studies, patient populations, indications, drugs) thereby incorporating wide sources of variability to provide adequate and robust prediction of outcomes based on the individual’s emerging data. Although methodological progress has been made, there is still more work to do on wider implementation and uptake. Lack of appropriate and consistent data standards, consensus on modeling approaches, adequacy in testing, and regulatory policies around the approval and use of such applications are some areas where progress is lacking.

### Clinical Utility Index (CUI)

Clinical utilization of a therapeutic intervention beyond regulatory approval is based on evidence of effectiveness in the real-world environment. Effectiveness trials are more pragmatic in nature that the study conditions are more clinically relevant than controlled efficacy trials performed for regulatory approval. The pharmacy and therapeutics (P&T) committees are charged with an important task to review the effectiveness of a new intervention to introduce into the formulary of a hospital or managed care organization. A recent workshop conducted by the Academy of Managed Care Pharmacy (AMCP) concluded that new evaluation methods and competencies are required for the P&T members with the availability of new data sources (AMCP Partnership Forum, 2020). A similar scenario in drug development teams, especially in the early clinical development, is benchmarking the product profile with other existing standard of care therapies and making a go/no-go decision for more expensive confirmatory clinical trials. The CUI is a product-level quantitative multi-attribute utility function used to make such complex decisions that require tradeoffs between multiple clinical attributes of an investigational therapy. Several examples of CUI in different therapeutic areas can be found in the references (Ouellet et al., 2009; Zhu et al., 2019). The CUI is analogous to other public health metrics such as number needed to treat (NNT), number needed to harm (NNH), and quality of life years (QALY) measures but provides a greater transparency in the evaluation of target product profile and improves the collaboration within the P&T committees to contribute to the relative weights of each of those attributes.

## Barriers of Implementation of M&S Tools for Clinic-Based Precision Dosing

The two important drivers for the success of precision dosing in clinic will be physicians and clinical pharmacists. In addition to the complex regulatory and healthcare system level barriers such as billing and infrastructure, one of the most critical barrier for the successful implementation of M&S tools for precision dosing is the dangerously low level of clinical pharmacology education in the medical community (Donnenberg et al., 2016). Clinical pharmacists are in a relatively better position with their skills in TDM, but there is a dire need to include pharmacometrics components into the curriculum to enhance the skill level. Clinical practice beyond drug approval is influenced by the guidelines from the societies of particular disease areas. The rise of M&S tools into clinical practice calls for a strategic collaboration between prominent pharmacometrics communities and the disease-specific societies for a seamless flow of knowledge transfer.

There is a small but realistic risk of over-enthusiasm while implementing and applying such model-based tools in clinical practice. Complicating a dosing regimen for wide therapeutic index drugs just to implement precision dosing could potentially result in more dosing errors or additional health care costs, causing more harm than benefit to the patient. The end goal of any approach is to provide safe and efficacious drug in a timely and cost-effective manner. The dosing scheme should also consider low- and middle-income countries where healthcare literacy may vastly differ from developed regions such as the U.S. or European countries.

## Future Directions

The need for precision dosing was recently weighed by the U.S. FDA in a public workshop. In their evaluation, at least 50% of 181 drugs approved between 2013–2017 qualified for precision dosing (Traynor, 2019). However, there is a dangerously low number of clinical pharmacists and physicians, who are the end-users, involved in pharmacometrics societies. It is important that the pharmacometrics community extend their influence beyond drug development and build bridges with clinical practitioners. There is also a need to standardize the data collection and formatting practices in TDM so that the data can readily be utilized in the process of Bayesian model updates and Bayesian forecasting. In this regard, the Institute of Medicine’s (IOM) proposal of Learning Health Systems (LHS) may provide an excellent infrastructure to bring research and practice together to enable the full potential of precision dosing efforts, leading to precision medicine (Chambers et al., 2016; Ramsey et al., 2017).

## Conclusion

Precision dosing should not be interpreted narrowly as the use of a fancy set of dose calculators. The clinical community must embrace the philosophy of pharmacometrics, which is a disciplined method for making decisions using current and historical scientific evidence. It is important that this paradigm shift in clinical practice is recognized by regulatory agencies, managed care organizations, payers, and academic institutions to make an orchestrated effort.

## Author Contributions

AC, BP, HC, and NG all wrote sections to the manuscript.

## Conflict of Interest

NG is an employee of GlaxoSmithKline.

The remaining authors declare that the research was conducted in the absence of any commercial or financial relationships that could be construed as a potential conflict of interest.

## References

[B1] AMCP Partnership Forum (2020). AMCP Partnership Forum: Principles for Sound Pharmacy and Therapeutics (P&T) Committee Practices: What’s Next? J. Manag. Care Spec. Pharm. 26 (1), 48–53. 10.18553/jmcp.2020.26.1.48 31880220 PMC10391133

[B2] Barker C. Standing J. F. Kelly L. E. Hanly Faught L. Needham A. C. Rieder M. J. (2018). Pharmacokinetic studies in children: recommendations for practice and research. Arch. Dis. Childhood 103 (7), 695–702. 10.1136/archdischild-2017-314506 29674514 PMC6047150

[B3] Chambers D. A. Feero W. G. Khoury M. J. (2016). Convergence of Implementation Science, Precision Medicine, and the Learning Health Care System: A New Model for Biomedical Research. JAMA 315 (18), 1941–1942. 10.1001/jama.2016.3867 27163980 PMC5624312

[B4] Charlesworth C. J. Smit E. Lee D. S. Alramadhan F. Odden M. C. (2015). Polypharmacy Among Adults Aged 65 Years and Older in the United States: 1988-2010. J. Gerontol. A Biol. Sci. Med. Sci. 70 (8), 989–995. 10.1093/gerona/glv013 25733718 PMC4573668

[B5] Cho S. Lau S. W. J. Tandon V. Kumi K. Pfuma E. Abernethy D. R. (2011). Geriatric Drug Evaluation: Where Are We Now and Where Should We Be in the Future? Arch. Intern Med. 171 (10), 937–940. 10.1001/archinternmed.2011.152 21606098

[B6] Collins F. S. Varmus H. (2015). A new initiative on precision medicine. N. Engl. J. Med. 372 (9), 793–795. 10.1056/NEJMp1500523 25635347 PMC5101938

[B7] Denson A. C. Mahipal A. (2014). Participation of the elderly population in clinical trials: barriers and solutions. Cancer Control 21 (3), 209–214. 10.1177/107327481402100305 24955704

[B8] Donnenberg V. S. Burris J. F. Wiernik P. H. Cohen L. J. Korth-Bradley J. M. (2016). How to Fix the Dangerous Lack of Clinical Pharmacology Education in the Medical Profession: The Generation of Core Entrustable Professional Activities in Clinical Pharmacology for Entering Residency. J. Clin. Pharmacol. 56, 1177–1179. 10.1002/jcph.748 27068769

[B9] Gad S. C. (2003). “Evaluation of Human Tolerance and Safety in Clinical Studies: Phase I and Beyond,” in Drug Safety Evaluation. Ed. Gad S. C.. 10.1002/0471462993.ch20

[B10] Kearns G. L. Abdel-Rahman S. M. Alander S. W. Blowey D. L. Leeder J. S. Kauffman R. E. (2003). Developmental pharmacology–drug disposition, action, and therapy in infants and children. N. Engl. J. Med. 349 (12), 1157–1167. 10.1056/NEJMra035092 13679531

[B11] Lau S. Cheung L. K. Chow D. (2015). “Application of Pharmacokinetics to Specific Populations: Geriatric, Obese, and Pediatric Patients,” in Applied Biopharmaceutics & Pharmacokinetics. Eds. Shargel L. Yu A. C. (New York, NY: McGraw-Hill), 7e.

[B12] Neely M. Jelliffe R. (2010). Practical, Individualized Dosing: 21st Century Therapeutics and the Clinical Pharmacometrician. J. Clin. Pharmacol. 50, 842–847. 10.1177/0091270009356572 20154293

[B13] Ouellet D. Werth J. Parekh N. Feltner D. McCarthy B. Lalonde R. (2009). The Use of a Clinical Utility Index to Compare Insomnia Compounds: A Quantitative Basis for Benefit–Risk Assessment. Clin. Pharmacol. Ther. 85, 277–282. 10.1038/clpt.2008.235 19078947

[B14] Peck R. W. (2018). Precision Medicine Is Not Just Genomics: The Right Dose for Every Patient. Annu. Rev. Pharmacol. Toxicol. 58 (1), 105–122. 10.1146/annurev-pharmtox-010617-052446 28961067

[B15] Ramsey L. B. Mizuno T. Vinks A. A. Margolis P. A. (2017). Learning Health Systems as Facilitators of Precision Medicine. Clin. Pharmacol. Ther. 101 (3), 359–367. 10.1002/cpt.594 27984650 PMC5309135

[B16] Sheiner L. B. Beal S. L. (1980). Evaluation of methods for estimating population pharmacokinetic parameters. I. Michaelis-menten model: Routine clinical pharmacokinetic data. J. Pharmacokinet. Biopharmaceut. 8, 553–571. 10.1007/bf01060053 7229908

[B17] Sheiner L. B. Beal S. L. (1981). Evaluation of methods for estimating population pharmacokinetic parameters II. Biexponential model and experimental pharmacokinetic data. J. Pharmacokinet. Biopharmaceut. 9, 635–651. 10.1007/bf01061030 7334463

[B18] Sheiner L. B. Beal S. L. (1983). Evaluation of methods for estimating population pharmacokinetic parameters. III. Monoexponential model: Routine clinical pharmacokinetic data. J. Pharmacokinet. Biopharmaceut. 11, 303–319. 10.1007/bf01061870 6644555

[B19] Sheiner L. B. (1997). Learning versus confirming in clinical drug development. Clin. Pharmacol. Ther. 61, 275–291. 10.1016/S0009-9236(97)90160-0 9084453

[B20] Shenoy P. Harugeri A. (2015). Elderly patients’ participation in clinical trials. Perspect. Clin. Res. 6 (4), 184–189. 10.4103/2229-3485.167099 26623388 PMC4640010

[B21] Traynor K. (2019). FDA examines precision dosing. Am. J. Health Syst. Pharm. 76 (24), 1999–2000. 10.1093/ajhp/zxz269 31789359

[B22] U.S. Department of Health and Human Services Food and Drug Administration. Center for drug evaluation and research (2007). Programs affecting safety and innovation in pediatric therapies. U.S. Government Printing Office Available at: https://www.govinfo.gov/content/pkg/CHRG-110hhrg41972/pdf/CHRG-110hhrg41972.pdf [Accessed 25th Nov 2019]

[B23] United States Census Bureau (2017). Older People Projected to Outnumber Children for First Time in U.S. History. Available at: https://www.census.gov/data/tables/2017/demo/popproj/2017-summary-tables.html.

[B24] Verstegen R. H. Ito S. (2019). The Future of Precision Medicine. Clin. Pharmacol. Ther. 106, 903–906. 10.1002/cpt.1622 31584718

[B25] Vinks A. A. Peck R. W. Neely M. Mould D. R. (2020). Development and Implementation of Electronic Health Record–Integrated Model-Informed Clinical Decision Support Tools for the Precision Dosing of Drugs. Clin. Pharmacol. Ther. 107, 129–135. 10.1002/cpt.1679 31621071

[B26] Wang Y. Zhu H. Madabushi R. Liu Q. Huang S.-M. Zineh I. (2019). Model-Informed Drug Development: Current US Regulatory Practice and Future Considerations. Clin. Pharmacol. Ther. 105, 899–911. 10.1002/cpt.1363 30653670

[B27] Zhu R. Poland B. Wada R. Liu Q. Musib L. Maslyar D. (2019). Exposure–Response-Based Product Profile–Driven Clinical Utility Index for Ipatasertib Dose Selection in Prostate Cancer. CPT Pharmacometr. Syst. Pharmacol. 8, 240–248. 10.1002/psp4.12394 PMC6482275 30762302

